# Immunoediting of stress-induced behaviors using cell-based technologies

**DOI:** 10.1192/j.eurpsy.2025.2032

**Published:** 2025-08-26

**Authors:** E. Markova, M. Knyazheva, E. Serenko, I. Savkin, O. Anikeeva

**Affiliations:** 1 Neuroimmunology Lab, State Research Institute of Fundamental and Clinical Immunology, Novosibirsk, Russian Federation

## Abstract

**Introduction:**

Negative consequences of acute and chronic stress caused by information overload, environmental problems, natural disasters, man-made disasters, military clashes, and acts of terrorism have become increasingly evident in recent decades. They can lead to a painful “breakdown” of socio-biological adaptation mechanisms, a decrease in adequacy in assessing the environment, and, as a consequence, the formation of disturbances in the psycho-emotional sphere, manifested in the development of behavioral disorders (aggression and depression). Therapy for behavioral disorders does not provide a complete cure, apparently due to the formation of a “vicious circle” that can only be broken by normalizing the regulatory relationship between the central nervous system and the immune system. One way to solve this problem is to develop new methods of therapy based on immunological approaches. The method of choice may obviously be immunotherapy with autologous immune cells with a certain, including in vitro modulated, functional activity.

**Objectives:**

The aim of the study was to investigate the possibility of obtaining a positive psychoneuroimmunomodulatory effect in aggressive and depressive-like animals by transplanting immune cells, the functional activity of which was in vitro modulated by a psychoactive substance (PAS).

**Methods:**

Male (CBAxC57Bl/6)F1 mice aged, in which aggressive or depressive-like states were formed under the influence of long-term social stress, were used as donors and recipients in the experiments. Immune cells for transplantation were obtained from a suspension of donor’s splenocytes, cultured in vitro with PAS (chlorpromazine or caffeine, respectively) and then intravenously administered to syngeneic recipients. In the control group of animals, cell preparation and transplantation were carried out under similar conditions, except that the latter were cultured without the presence of PAS. In recipients the parameters of the nervous and immune systems functional activity were recorded.

**Results:**

Transplantation of in vitro PAS-modulated splenocytes to recipients with stress-induced behavior patterns resulted in targeted changes in their motor and exploratory activity in the Open field test, motor activity in the Porsolt test, and decreased anhedonia in recipients with a depressive-like phenotype, recorded against the background of changes in the content of a number of pro- and anti-inflammatory cytokines IL-1β, IL-2, IL-4, IL-6, IL-10, IFNγ, TNFα in pathogenetically significant brain structures. In recipients, a positive change in the parameters of the immune system functional activity were also revealed.

**Conclusions:**

The possibility of immunoediting stress-induced forms of behavior by transplanting in vitro PAS-modulated immune cells, which have a positive psychoneuroimmunomodulatory effect in the body of syngeneic recipients, has been demonstrated.

**Disclosure of Interest:**

None Declared

